# Prevalence of Vitamin D Deficiency in Type 2 Diabetes Mellitus Patients: A Cross-Sectional Study

**DOI:** 10.7759/cureus.38952

**Published:** 2023-05-12

**Authors:** Gopika S Vijay, Swati Ghonge, Sai Mahesh Vajjala, Deepu Palal

**Affiliations:** 1 Community Medicine, Dr. D. Y. Patil Medical College Hospital and Research Center, Dr. D. Y. Patil Vidyapeeth, Pune, IND

**Keywords:** prevalence, non-communicable disease, obesity, prevention, vitamin-d deficiency, diabetes mellitus type 2

## Abstract

Background

Type 2 diabetes mellitus (T2DM) is one of the most common non-communicable diseases, causing a high mortality rate globally. Vitamin D deficiency has been reported as a rising pandemic. Vitamin D levels have been found to be associated with obesity and insulin resistance. However, there is not much research done to study the various factors associated with the association between vitamin D levels and diabetes mellitus in the Indian population.

Objective

The objective of this study is to estimate the prevalence of vitamin D deficiency in T2DM patients and find the factors associated with vitamin D levels among type 2 diabetes mellitus patients.

Materials and methods

A cross-sectional analytical study was planned and done in the Urban Health Training Centre of Dr. D.Y. Patil Medical College. The sample size was calculated using published data on prevalence. Data from 116 T2DM patients were collected using a questionnaire about their socio-economic status, dietary patterns, outdoor activities, exercise, drug and supplement intake, occupation, and symptoms, which was filled out by the participants after written informed consent. Levels of serum vitamin D were estimated from the blood samples taken from the participants. Statistical analysis was done using MedCalc software.

Results

Vitamin D deficiency was found in 86 (74.14%) out of 116 diabetic patients. There were 63 males, and 71.43% of them had lower-than-normal vitamin D levels. The number of female participants was 53, and 77.36% were found to suffer from vitamin D deficiency. There were 88 obese participants, and only 22.73% were found to have sufficient levels of vitamin D.

Conclusion

The results depict a high prevalence of vitamin D deficiency in type 2 diabetes mellitus patients. Regular supplementation of vitamin D for diabetic patients can prevent them from developing any further complications. Increasing awareness about a healthy lifestyle, a proper diet, adequate sunlight, and exercise can help keep most non-communicable diseases at bay. Additional studies should be done to understand the pathophysiology better, which can aid in preventing diseases in the beginning stages of development.

## Introduction

Various non-communicable diseases have become global pandemics because of the non-sustainable way of life followed by the general population. The various complications associated with these disorders have led to decreased life expectancy. In today’s world, people tend to feel an urgency to keep up with their day-to-day activities. This happens at the cost of their own health. Various deficiencies are seen due to the decreased intake of nutrient-rich foods. In addition to this, the majority of people spend more time indoors, which results in decreased sunlight exposure.

In 2019, diabetes contributed to 1.5 million deaths globally and was the ninth leading cause of death [1]. Type 2 diabetes mellitus (T2DM) is a chronic metabolic disorder in which insulin resistance is seen and the normal metabolism of carbohydrates, fat, and protein is disturbed, leading to various microvascular and macrovascular complications. The microvascular complications of diabetes cause kidney disease and blindness, which might lead to amputations. Apart from those, heart attacks and strokes are macrovascular complications associated with diabetes [2].

Vitamin D deficiency has become a global pandemic, including in areas that receive adequate sunlight [3]. The sun's ultraviolet B (UVB) rays cause the skin to produce 7-dehydrocholesterol, a fat-soluble vitamin. 7-dehydrocholesterol is converted to pre-vitamin D3, which is then converted to vitamin D3 (cholecalciferol) by UVB rays. Vitamin D3 binds to the vitamin D-binding protein (DBP). The primary circulating form of vitamin D, 25-hydroxyvitamin D (25-OHD), is then created in the liver. The 25-OHD is absorbed by the kidneys and converted to 1,25(OH)2D (1,25 dihydroxyvitamin D), the active form of vitamin D. Numerous tissues have vitamin D receptors (VDR) that 1,25(OH)2D binds to [4].

Vitamin D is used in developing and maintaining the skeletal system by regulating serum calcium and serum phosphorous levels. Hence, deficiency of this vitamin can lead to skeletal manifestations, the most common being osteomalacia and osteoporosis in the adult group and rickets among children. Vitamin D deficiency is also known to lead to depression, cancer, and heart disease [3].

Obesity and overweight are abnormal or excessive fat accumulations that put one's health at risk. According to the World Health Organization, globally, more than 1.9 billion adults were overweight in 2016, of whom 650 million were obese [5]. The increased consumption of unhealthy processed foods, which are high in fat and sugar, along with the decreased activity by the population, might be the most probable reason for the rise in the prevalence of obesity all around the world. A significant risk factor for T2DM is obesity [5]. Additionally, people with higher BMIs have significantly lower vitamin D levels [3].

Vitamin D levels indirectly influence insulin secretion by regulating calcium flux through the cell membrane and intracellular calcium [4]. It has also been proposed that the pancreatic tissue and various cell-type immune systems express VDR [4]. According to past studies, VDR gene polymorphisms influence the activity of the VDR protein [6]. The genetic polymorphisms in the VDR, which altered calcium metabolism, adipocyte function, insulin release, and cytokine production, had a substantial impact on the development of type 2 diabetes mellitus [6]. A strong association between vitamin D3 and insulin resistance was found in a previous cross-sectional study of five periods in the NHANES (National Health and Nutrition Examination Survey) database. Subgroup analyses, however, showed that this correlation varied between people and races [7].

Furthermore, studies have shown that vitamin D levels in obese individuals are lower [8]. Various theories regarding the correlation were proposed. Some suggest that vitamin D is sequestered within adipose tissue [8]. Others proposed that obese individuals exhibit a negative feedback mechanism apart from receiving reduced sunlight exposure [8]. The last theory explained that the low levels of vitamin D are due to volumetric dilution [8-9].

Previous studies conducted in India, as well as in other countries, have shown that vitamin D levels have an influence on the pathogenesis of T2DM. However, relatively few research has been conducted to determine the actual burden of vitamin D deficiency among diabetic patients in India. This study was aimed at estimating the prevalence of vitamin D deficiency in T2DM patients, finding any other factors that may be associated with vitamin D levels among T2DM patients, and increasing awareness among T2DM patients regarding the importance of proper nutrition, a healthy lifestyle, and adequate sunlight exposure.

## Materials and methods

A cross-sectional study was conducted among adults with type 2 diabetes mellitus who were attending the general OPD in the Urban Health Training Centre, the urban field practice area of the Department of Community Medicine of Dr. D.Y. Patil Medical College, Pune, Maharashtra, India. The study was done for two months, from July 7, 2022 to September 7, 2022. The required approval from the Institutional Ethics Sub-Committee was obtained before starting the study, and the reference number for the same is I.E.S.C./109/2022. All diagnosed T2DM patients aged 18 and above who were willing to participate were included in the study, considering the feasibility and the patient profile of the urban health center. Patients with pre-existing renal or parathyroid disorders were excluded.

Sample size and sampling technique

The prevalence of vitamin D deficiency in various previously conducted studies was found to range between 70% and 100% [10]. Considering an estimated prevalence of vitamin D deficiency in T2DM of 50% and using Open-Epi software, at a precision of 10%, a 95% confidence level, and a power of 80%, the sample size comes to 96. We have considered a 15% non-response rate, and the final sample size was 113. However, we have collected data from 116 participants. Due to time and resource constraints, the consecutive sampling technique was utilized. All eligible, consenting participants were included in the study.

The procedure and its importance were explained to all the participants. Participants were informed, and written consent was obtained. Interviews were conducted using a structured questionnaire that was self-designed and pre-tested. The structured questionnaire contained details about socio-economic status, dietary patterns, outdoor activities, exercise, drug and supplement intake, occupation, and any symptoms. The height and weight of each participant were recorded using calibrated instruments and trained staff, and the BMI was calculated and classified according to Asian standards. Random blood sugar levels were measured by a single glucometer throughout the study period. Instruments such as measuring tapes, weighing scales, sphygmomanometers, and glucometers were all validated before being used for the study. Five milliliters of blood were collected with aseptic precautions in plain bulbs and sent for vitamin D levels. The main diagnostic lab was <5 km away, and a proper cold chain was maintained in transporting the samples. Serum vitamin D levels were estimated quantitatively using a Roche kit developed by Roche Diagnostics, Mannheim, Germany. Vitamin D deficiency is defined as vitamin D levels less than 20 ng/ml, insufficient levels as levels between 20 ng/ml and 29 ng/ml, and sufficient or normal levels as levels equal to or above 30 ng/ml.

The data were entered into Microsoft Excel (Microsoft® Corp., Redmond, WA) and analyzed using MedCalc software (MedCalc Software, Ltd., Belgium). Descriptive statistics are presented by frequency and percentage. Continuous variables were depicted by mean, standard deviation, median, or range after assuming normality using the Shapiro-Francia test. Appropriate tests of significance, like the Chi-square test for categorical data and the t-test, were used, keeping significance at p<0.05.

## Results

A total of 116 participants’ information was used for analysis. A detailed demographic presentation is given in Table 1. Almost three-fourths of the participants were over 45 years old. There was not much difference among the male and female T2DM patients across different categories. Obesity was observed in most (75.86%) of the study sample. A good number of the T2DM patients were graduates (66.98%). Pure vegetarians were comparatively few (29.31%). Anything other than basic indoor exercises and gymnasium activities was considered an outdoor activity. A smaller number of the participants were doing outdoor activities (35.34%), but most of them, 75/116 (64.66%), were doing exercises. Exercises included aerobic exercises, cycling, walking, and any other related activities of more than 30 minutes duration. It was good to observe that almost four-fifths (78.45%) had their sugar levels under control. The status of diabetes control was assessed using any HbA1c report within the past three months; if not available, random blood sugar levels were checked for the participants. While a majority of them were on oral hypoglycemic agents (93.2%), a few (6.8%) were being treated with insulin preparations. While the majority of the participants had their vitamin D levels insufficient (39.66%), deficient and sufficient were found in 40 (34.48%) and 30 (25.86%) participants, respectively. Almost half the participants were hypertensive 56 (48.28%).

**Table 1:** Demographic characteristics of the study population

	Male, n (%)	Female, n (%)	Total, n (%)
Age category (years)			
<35	5 (7.94)	2 (3.77)	7 (6.03)
36–45	14 (22.22)	7 (13.21)	21 (18.1)
46–60	19 (30.16)	20 (37.74)	39 (33.62)
>60	25 (39.68)	24 (45.28)	49 (42.24)
Total	63 (100)	53 (100)	116 (100)
Body mass index			
Underweight	1 (1.59)	1 (1.89)	2 (1.72)
Normal	6 (9.52)	5 (9.43)	11 (9.48)
Overweight	9 (14.29)	6 (11.32)	15 (12.93)
Obese	47 (74.6)	41 (77.36)	88 (75.86)
Total	63 (100)	53 (100)	116 (100)
Education			
Below primary	4 (6.45)	4 (9.09)	8 (7.55)
Primary	3 (4.84)	4 (9.09)	7 (6.6)
Secondary	8 (12.9)	3 (6.82)	11 (10.38)
High school	5 (8.06)	4 (9.09)	9 (8.49)
Graduate	42 (67.74)	29 (65.91)	71 (66.98)
Total	62 (100)	44 (100)	106 (100)
Diet			
Vegetarian	18 (28.57)	16 (30.19)	34 (29.31)
Mixed (non-vegetarian)	45 (71.43)	37 (69.81)	82 (70.69)
Total	63 (100)	53 (100)	116 (100)
Outdoor activities			
Yes	24 (38.1)	17 (32.08)	41 (35.34)
No	39 (61.9)	36 (67.92)	75 (64.66)
Total	63 (100)	53 (100)	116 (100)
Exercise			
Yes	45 (71.43)	30 (56.6)	75 (64.66)
No	18 (28.57)	23 (43.4)	41 (35.34)
Total	63 (100)	53 (100)	116 (100)
Sugar levels controlled or not			
Controlled	49 (77.78)	42 (79.25)	91 (78.45)
Not controlled	14 (22.22)	11 (20.75)	25 (21.55)
Total	63 (100)	53 (100)	116 (100)
Drug history in past 6 months			
Insulin	3 (5.26)	4 (8.7)	7 (6.8)
Oral hypoglycemic agents	54 (94.74)	42 (91.3)	96 (93.2)
Total	57 (100)	46 (100)	103 (100)
Vitamin D levels			
Deficient	20 (31.75)	20 (37.74)	40 (34.48)
Insufficient	25 (39.68)	21 (39.62)	46 (39.66)
Sufficient/optimal	18 (28.57)	12 (22.64)	30 (25.86)
Total	63 (100)	53 (100)	116 (100)
Hypertension			
Yes	28 (44.44)	28 (52.83)	56 (48.28)
No	35 (55.56)	25 (47.17)	60 (51.72)
Total	63 (100)	53 (100)	116 (100)

Various factors were assessed for independence and association with vitamin D levels and are presented in Table 2. A statistically significant association was not found for any factors assessed, namely age, sex, BMI, education, diet, outdoor activities, exercise, controlled sugar levels, hypertension, and dyslipidemia. Row percentages are presented to compare the variables across the deficient, insufficient, and sufficient categories. Upon appropriate statistical testing, none of the variables were significantly associated. This is partly due to the limited sample size and also because there was not a big difference in the proportions across the categories.

**Table 2:** Categorical representation of vitamin D levels of the study sample

Vitamin D status	Deficient, n (%)	Insufficient, n (%)	Sufficient/optimal, n (%)	Total, n (%)	Statistical test of significance
Age (years)					Chi squared (6) = 7.543, p = 0.2736
<35	3 (7.5)	3 (6.52)	1 (3.33)	7 (6.03)	
36–45	10 (25)	9 (19.57)	2 (6.67)	21 (18.1)	
46–60	12 (30)	18 (39.13)	9 (30)	39 (33.62)	
>60	15 (37.5)	16 (34.78)	18 (60)	49 (42.24)	
Total	40 (34.48)	46 (39.66)	30 (25.86)	116 (100)	
Sex					Chi squared (2) = 0.691, p = 0.7079
Male	20 (50)	25 (54.35)	18 (60)	63 (54.31)	
Female	20 (50)	21 (45.65)	12 (40)	53 (45.69)	
Total	40 (34.48)	46 (39.66)	30 (25.86)	116 (100)	
Body mass index					Fisher’s exact p = 0.7237
Underweight	0 (0)	1 (2.17)	1 (3.33)	2 (1.72)	
Normal	2 (5)	5 (10.87)	4 (13.33)	11 (9.48)	
Overweight	5 (12.5)	5 (10.87)	5 (16.67)	15 (12.93)	
Obese	33 (82.5)	35 (76.09)	20 (66.67)	88 (75.86)	
Total	40 (34.48)	46 (39.66)	30 (25.86)	116 (100)	
Education					Fisher’s exact p = 0.0598
Below primary	4 (10.53)	2 (4.76)	2 (7.69)	8 (7.55)	
Primary	0 (0)	5 (11.9)	2 (7.69)	7 (6.6)	
Secondary	5 (13.16)	0 (0)	6 (23.08)	11 (10.38)	
High school	3 (7.89)	4 (9.52)	2 (7.69)	9 (8.49)	
Graduate	26 (68.42)	31 (73.81)	14 (53.85)	71 (66.98)	
Total	38 (32.76)	42 (36.21)	26 (22.41)	106 (100)	
Diet					Chi squared (2) = 0.607, p = 0.7382
Vegetarian	10 (25)	15 (32.61)	9 (30)	34 (29.31)	
Mixed (non-vegetarian)	30 (75)	31 (67.39)	21 (70)	82 (70.69)	
Total	40 (34.48)	46 (39.66)	30 (25.86)	116 (100)	
Exercise					Fisher’s exact p=0.291
Less than 1 hour	0 (0)	2 (5.56)	2 (11.11)	4 (3.45)	
More than 1 hour	22 (100)	34 (94.44)	16 (88.89)	72 (62.07)	
Total	22 (28.95)	36 (47.37)	18 (23.68)	76 (65.52)	
Outdoor activities					Chi squared (2) = 4.504, p = 0.1052
Yes	15 (37.5)	20 (43.48)	6 (20)	41 (35.34)	
No	25 (62.5)	26 (56.52)	24 (80)	75 (64.66)	
Total	40 (34.48)	46 (39.66)	30 (25.86)	116 (100)	
Hypertension					Chi squared (2) = 0.082, p = 0.9596
Yes	20 (50)	22 (47.83)	14 (46.67)	56 (48.28)	
No	20 (50)	24 (52.17)	16 (53.33)	60 (51.72)	
Total	40 (34.48)	46 (39.66)	30 (25.86)	116 (100)	
Sugar levels controlled or not					Chi squared (2) = 2.583, p = 0.2749
Yes	28 (70)	38 (82.61)	25 (83.33)	91 (78.45)	
No	12 (30)	8 (17.39)	5 (16.67)	25 (21.55)	
Total	40 (34.48)	46 (39.66)	30 (25.86)	116 (100)	
Drug history in past 6 months					Fisher’s exact p=0.2729
Insulin	3 (8.33)	4 (9.76)	0 (0)	7(6.8)	
Oral hypoglycemic agents	33 (91.67)	37 (90.24)	26 (100)	96(93.2)	
Total	36 (34.9)	41 (39.8)	26 (25.2)	103(100)	
Dyslipidemia					Chi-squared (2) = 0.49, p=0.782
Yes	6 (15)	7 (15.22)	3 (10)	16 (13.79)	
No	34 (85)	39 (84.78)	27 (90)	100 (86.21)	
Total	40 (34.48)	46 (39.66)	30 (25.86)	116 (100)	
Exercise status					Chi-squared (2) = 4.54, p=0.103
Exercise regularly	22 (55)	35 (76.1)	18 (60)	75 (64.7)	
Do not exercise regularly	18 (45)	11 (23.9)	12 (40)	41 (35.3)	
Total	40 (34.48)	46 (39.66)	30 (25.86)	116 (100)	
Diabetes mellitus duration					Chi-squared (2) = 5.49, p=0.064
Three years and below	23 (57.5)	27 (58.7)	10 (33.3)	60 (51.7)	
More than three years	17 (42.5)	19 (41.3)	20 (66.7)	56 (48.2)	
Total	40 (34.48)	46 (39.66)	30 (25.86)	116 (100)	

Also, associations for symptoms with various levels of vitamin D were tested and presented in Table 3. The participants inquired about issues like bone pain, dental issues, fatigue, muscle pain, and others. Only fatigue as a symptom was associated independently with vitamin D categories (insufficient and deficient had higher proportions of fatigue). Dental problems, muscle pain, bone pain, and other symptoms (other complications of diabetes that were less prevalent, e.g., foot ulcers; some of the study population even said they had breathlessness in the recent past) were not statistically different among the vitamin D categories. Approximately equal proportions of deficient, insufficient, and sufficient category T2DM patients either had or did not have these symptoms.

**Table 3:** Association of symptoms with vitamin D levels

Vitamin D category	Deficient, n (%)	Insufficient, n (%)	Sufficient/optimal, n (%)	Total, n (%)	Statistical test of significance
Bone pain					
Present	22 (42.3%)	19 (36.5%)	11 (21.2%)	52 (44.8%)	Chi-squared (2) = 2.712, p = 0.2577
Absent	18 (28.1%)	27 (42.2%)	19 (29.7%)	64 (55.2%)	
Total	40 (34.5%)	46 (39.7%)	30 (25.9%)	116 (100%)	
Dental problem					
Present	18 (38.3%)	18 (38.3%)	11 (23.4%)	47 (40.5%)	Chi-squared (2) = 0.555, p = 0.7578
Absent	22 (31.9%)	28 (40.6%)	19 (27.5%)	69 (59.5%)	
Total	40 (34.5%)	46 (39.7%)	30 (25.9%)	116 (100%)	
Fatigue					
Present	25 (41.7%)	25 (41.7%)	10 (16.7%)	60 (51.7%)	Chi-squared (2) = 6.05, p = 0.0485
Absent	15 (26.8%)	21 (37.5%)	20 (35.7%)	56 (48.3%)	
Total	40 (34.5%)	46 (39.7%)	30 (25.9%)	116 (100%)	
Muscle pain					
Present	22 (44%)	20 (40%)	8 (16%)	50 (43.1%)	Chi-squared (2) = 5.616, p = 0.0603
Absent	18 (27.3%)	26 (39.4%)	22 (33.3%)	66 (56.9%)	
Total	40 (34.5%)	46 (39.7%)	30 (25.9%)	116 (100%)	
Any other symptoms					
Present	6 (30%)	7 (35%)	7 (35%)	20 (17.2%)	Chi-squared (2) = 1.053, p = 0.5906
Absent	34 (35.4%)	39 (40.6%)	23 (24%)	96 (82.8%)	
Total	40 (34.5%)	46 (39.7%)	30 (25.9%)	116 (100%)	

## Discussion

In the present study, we found that 74.14% (86/116) of participants with T2DM had less than sufficient levels of vitamin D, which includes 34.48% in the deficient category and 39.66% in the insufficient category.

In a study done in the southern part of India by Palazhy et al., among the diabetic population (which included 4628 subjects), they found that 71.4% were vitamin D deficient, 15% were vitamin D insufficient, and 13.6% had normal vitamin D levels [11]. In another study conducted by Prasad et al. among the diabetic population residing in Karnataka, India, it was found that the prevalence of vitamin D deficiency in their diabetic study subjects was 78.2%, which is similar to the results in our study [12]. According to a retrospective case-control study conducted in India, vitamin D deficiency and insufficiency were found in 32.1% and 34.6% of patients with T2DM, respectively [13]. A study conducted in the northern states of India found that vitamin D deficiency was reported in 91.1% of subjects with diabetes. In this particular study, the study subjects were newly diagnosed youth-onset diabetes mellitus patients [14].

According to a study done among the population group in Lagos, Nigeria, the prevalence of vitamin D deficiency was found to be 63.2% in T2DM patients, which is in a similar range [15]. The prevalence of vitamin D deficiency and insufficiency was found to be 38.4% and 21.9%, respectively, in another study conducted in a referral hospital in Kenya with only T2DM patients as the study group [16]. The majority of individuals in research conducted at the King Faisal University Health Center in Saudi Arabia who had T2DM had vitamin D levels that were below normal, which demonstrated a similar pattern to the current study [17]. All the studies mentioned above, including the current study, indicate that the prevalence of vitamin D deficiency is significantly higher in T2DM patients in India and other parts of the world.

Among the 116 participants in the study, 63 (54.3%) were male and 53 (45.7%) were female. A statistically significant association between vitamin D levels and the gender of the participants could not be found in the present study. This was similar to the result seen in the retrospective study conducted in the southern part of India, where, on comparing the genders, it was seen that the percentage of males and females with these conditions was similar [11]. Similar results were reported in a cross-sectional study of participants from the UAE, India, Pakistan, and Egypt that investigated the relationship between vitamin D insufficiency in obesity and a number of other metabolic variables. Gender and vitamin D levels have not been proven to be significantly correlated. Nevertheless, it has been found that age and vitamin D levels are positively associated [8]. Most of the current study participants belong to the age group of 66 and above. The prevalence of vitamin D deficiency in this age group is 58.33%. Where the participants belonging to the age category of 35-46 years have a significantly higher prevalence of vitamin D deficiency (90.48%), this is contrary to the finding of the multiethnic cohort study [8]. The result reported in this study could be due to the changes in lifestyle and eating habits between the two age groups. The sedentary lifestyle observed by the middle-aged group compared to those reaching retirement age could also be a factor.

Most of the participants (88/116) in the current study belong to the obese category (75.86%) according to the Asian standards of BMI classification. Amongst them, the prevalence of vitamin D deficiency and insufficiency was comparatively higher (37.5% and 39.7%, respectively). Only 22.7% (20/88) of obese participants had sufficient vitamin D levels. No statistically significant difference could be seen. The incidence of vitamin D levels in obese patients was examined retrospectively in two ambulatory clinics in South Florida, and the results exhibited a strong link between vitamin D deficiency and obesity (p<0.05) [18]. Hypertension and high cholesterol are the other two main non-communicable diseases seen in the Indian population, apart from type 2 diabetes mellitus. The current investigation found no conclusive evidence of a relationship between hypertension and vitamin D levels (p-value =0.9596). On the contrary, in a study done in a tertiary care center in South India, hypertension was associated with vitamin D deficiency [19]. However, the current study has only 56 hypertensive participants and 16 participants whose cholesterol levels were deranged. Therefore, it is difficult to conclude that the comorbidities are associated with vitamin D levels.

When people with diverse diets were compared, there was no significant variance in their levels of vitamin D (p=0.7382). This implies that diet has no role in the vitamin D status of an individual. However, in a study done in 2012 on 176 healthy subjects, it was concluded that vitamin D in non-vegetarians was significantly lower compared to vegetarians [20]. The current investigation shows no significant correlation between glycemic status and vitamin D levels when taking glycemic control into account. (p=0.2749). This contradicts a study conducted in Duhok, where vitamin D levels were considered significantly lower in participants with poor glycemic control [21].

Concerning exercise, there has been no significant association with vitamin D levels in the current study (p=0.103). Most likely, such results might be because the participant exercises indoors. Also, a fair number of people prefer to exercise in the evening after the end of their day-to-day activities. This results in minimum sun exposure. More than 50% of the subjects in this study who had vitamin D insufficiency reported experiencing fatigue. Fatigue is significantly associated with vitamin D deficiency (p=0.0485). Nearly half of the participants who had vitamin D deficiency had symptoms of pain in their bones and muscles and suffered from dental problems. This implies that vitamin D is responsible for maintaining the normal well-being of an individual.

Strengths and limitations

The study has provided documentation of the vitamin D status of the type 2 diabetes mellitus population in an urban setting in Maharashtra, India. Data were accurately collected by trained staff in a timely manner, and a uniform, standardized testing technique was used. Also, none of the individuals took vitamin D supplementation, demonstrating that the results were unaffected by the use of additional supplements. However, the study could not depict the relationship between vitamin D and T2DM as this cross-sectional study was done only in T2DM patients. Moreover, the majority of the individuals belonged to the overweight category; hence, the cause of the high prevalence of vitamin D deficiency cannot be predicted. Also, the study was conducted on a small group of people located in the urban setting of Maharashtra; hence, it cannot indicate the status of the entire Indian population.

## Conclusions

Vitamin D deficiency is a worldwide pandemic in the present day. It has been noticed that even in the tropical regions of the world, where adequate sunlight exposure is present, vitamin D deficiency is still prevalent. T2DM is the most common non-communicable disease in the world. The lifestyle adopted by the populace at present is adversely affecting the health situation. Obesity and overweight influence diabetes mellitus as well as vitamin D levels, according to present and previous studies.

The high rate of vitamin D insufficiency in the research population raises the possibility that diabetes may have an impact on the body's regulation of vitamin levels. The study provided an opportunity to create awareness among the general population about the health situation. A significant prevalence of vitamin D deficiency was observed in diabetic patients. This could imply that adequate supplementation of vitamin D in deficient populations could aid in the primary prevention of diabetes, along with other lifestyle modifications. Advocating for a healthy way of living can be the most important method to prevent the development of such grave diseases in the beginning. A healthy way of living includes a lifestyle with good outdoor exposure to sunlight, a proper diet, and exercise. Conducting health awareness programs, camps, and personal counseling at clinical visits would help the healthcare system achieve and maintain the well-being of T2DM patients and the general population.
